# Neurodevelopmental Disorders and the Mystery of the Genes Involved: A Case Report of a BICRA Heterozygous Mutation Identified in Autism Spectrum Disorder

**DOI:** 10.7759/cureus.66442

**Published:** 2024-08-08

**Authors:** María A Gratacós Arenas, Carolina Soler Portilla, Simón Carlo, Norma J Arciniegas

**Affiliations:** 1 Pediatrics, Centro Medico Episcopal San Lucas, Ponce, PRI; 2 Pediatrics, Ponce Health Sciences University, Ponce, PRI; 3 Genetics/Pediatrics, Mayagüez Medical Center, Mayagüez, PRI; 4 Biochemistry, Pediatrics, and Psychiatry, Ponce Health Sciences University, Ponce, PRI

**Keywords:** baf complex, neurodevelopmental disorders, bicra mutation, autism spectrum disorder, autism

## Abstract

Pathogenic variants in the BRD4 interacting chromatin remodeling complex associated protein (BICRA) are linked to BICRA-related neurodevelopmental disorders. These disorders are characterized by developmental delay, intellectual disability, and dysmorphic facial features, along with behavioral abnormalities, poor growth, vision abnormalities, and feeding difficulties. We present the case of a three-year-old male diagnosed with autism spectrum disorder (ASD), developmental speech delay, and epilepsy. Whole exome sequencing with copy number variant (CNV) analysis revealed a heterozygous variant of uncertain significance in the BICRA gene (c.1246G>C, p.Ala416Pro). This case report aims to highlight a gene associated with BICRA-related neurodevelopmental disorders that is rarely described in ASD patients. Further research is crucial to explore the role of chromatin remodeling in the etiology and development of ASD.

## Introduction

The BRD4 interacting chromatin remodeling complex associated protein (BICRA) is a protein-coding gene on chromosome 19q13.33 involved in the positive regulation of DNA transcription [1]. The protein encoded by this gene is a member of the SWItch/sucrose nonfermentable chromatin remodeling complex (SWI/SNF) complex, known as the BRG1- or BRM-associated factor (BAF) complex. The BAF complex enables transcriptional activation by modifying DNA-histone interactions in a nucleosome in an ATP-dependent manner. This mechanism promotes change to the chromatin structure and is critical to modulating gene expression.

In addition to its role in transcriptional activation, the BAF complex can also direct cell fate determination by forming cell type-specific complexes through different subunit combinations, as suggested by Sokpor et al. [2]. Further research has revealed that in addition to regulating gene expression, the BAF complex also plays a role in guiding crucial developmental processes and cognitive functions. For example, Ronan et al. demonstrate chromatin remodeling's importance in the development of neural circuits, as it directly influences neural plasticity and cognitive functions [3]. This study also links chromatin remodeling to developmental disorders, such as autism spectrum disorder (ASD) and intellectual disabilities, as well as mental health disorders like schizophrenia and depression, which can further affect cognitive functions. These findings provide a broader insight for understanding the complex mechanisms of BICRA within the chromatin remodeling domain.

Three BAF complexes have been identified: the Polybromo-associated BAF complex (PBAF), the canonical BAF complex (cBAF), and, most recently, the non-canonical BAF complex (ncBAF) or GBAF, named after its subunits (GLTSCR1/1L) [4]. BICRA, previously known as the glioma tumor suppressor candidate region gene 1 (GLTSCR1), is a crucial protein defining the ncBAF complex and is indispensable for chromatin remodeling. Recent evidence has found that mutations in the BICRA gene and the ncBAF-complex are associated with neurodevelopmental disorders, such as SWI/SNF-related neurodevelopmental disorders (SSRIDD). For example, Coffin-Siris syndrome, a condition within the SSRIDD category, is characterized by developmental disability, coarse facial features, and fifth-digit/nail hypoplasia [5].

We present the case of a three-year-old male diagnosed with ASD, developmental speech delay, and epilepsy. Whole exome sequencing with copy number variant (CNV) revealed a heterozygous variant of uncertain significance in the BICRA gene (c.1246G>C, p.Ala416Pro). This case report highlights the importance of further investigating the role of chromatin remodeling in the etiology and development of ASD. It also suggests that BICRA may be a new potential target for therapy, but more studies are needed to confirm the association between BICRA and ASD.

## Case presentation

The patient was born at full term of 40 weeks of gestation without immediate complications to unrelated parents of Puerto Rican descent. During the pregnancy, the patient's mother experienced influenza A+B and hyperemesis gravidarum, for which she was treated with Pepcid. Additionally, the mother was a victim of domestic abuse during the pregnancy. Despite these risk factors, no concerning symptoms were observed until the patient was hospitalized for an afebrile tonic-clonic seizure at five months old. His mother reports he had a second seizure episode a month later, for which levetiracetam was initiated. The medication was removed shortly after due to poor tolerance and an unremarkable electroencephalogram (EEG). At six months old, a geneticist evaluated the patient due to a positive screening test for methylmalonic aciduria and recurring seizures. Subsequent tests, including urine organic acids, lactic acid, pyruvic acid, ammonia, acylcarnitine, and carnitine levels, were performed to assess for metabolic and immune biomarkers associated with ASD, given the maternal history of infection and stress during pregnancy. All results were within normal limits.

By 18 months, the patient began showing evidence of hypotonia, alongside speech and language delay. His mother also reported poor eye contact, selective behaviors with food and toys, and repetitive hand movements. Family history includes maternal first cousins with ASD. A post-natal oligonucleotide single nucleotide polymorphism (SNP) chromosomal microarray was conducted to explore the patient's developmental disorder presentation. However, findings revealed no detectable CNVs or regions of homozygosity. The patient consequently underwent the fragile X syndrome (FXS) genetic test, confirming an adequate number of CGG repeats at the fragile X locus. Although these findings rule out fragile X based on the absence of expanded alleles, the test does not exclude fragile X as a possibility of other genetic variations, such as point mutations or gene rearrangements. Nevertheless, the patient was diagnosed with ASD and is currently undergoing therapy for speech, sensory integration, psychological support, and dysphagia. However, a whole exome sequence with CNV was performed to establish this patient's biological diagnosis further. Although results did not identify any pathogenic variants associated with the patient's clinical symptoms, the test identified a heterozygous variant of uncertain significance in the BICRA gene (c.1246G>C, p.Ala416Pro), as can be seen in Table 1. Variants in this gene are associated with BICRA-related neurodevelopmental disorders. These findings suggest further investigation regarding this variant's clinical significance and potential implications may be needed.

**Table 1:** Variant assessment of BICRA c1246G>C (p.Ala416Pro) BICRA c1246G>C (p.Ala416Pro) is classified as a missense variant of uncertain significance (VUS). This variant is associated with BICRA-related neurodevelopmental disorders.

Test	In range
Variant 1	
Gene	BICRA
Variant	c.1246G>C (p.Ala416Pro)
Zygosity	Heterozygous
Clinical relevance	Variant of uncertain significance
Inheritance	Autosomal dominant, unknown familiar inheritance
Associated phenotype	BICRA-related neurodevelopmental disorder

## Discussion

This study presents the case of a three-year-old male diagnosed with ASD, developmental speech delay, and epilepsy, with a heterozygous variant of uncertain significance (c.1246G>C, p.Ala416Pro) in the BICRA gene. The BICRA gene c.1246G>C (p.Ala416Pro) mutation has yet to be described in the literature. There are still many unanswered questions regarding neurodevelopmental disorders and their underlying biological causes.

The DSM-V defines neurodevelopmental disorders as a group of disorders characterized by developmental deficits, manifesting early in life, that produce impairments of personal, social, academic, and occupational functioning. These neurodevelopmental disorders are influenced by both genetic and environmental factors. A better understanding of the two-hit model and the variety of genetic mutations identified in these disorders can reveal genotype-phenotype correlations that could help monitor the disorders' progress, anticipate complications, and identify additional treatment options [6].

ASD is a multifactorial disorder significantly influenced by genetics. Family and twin studies have reported a high heritability in ASD, with identical twin studies showing a concordance rate of 70-90% [7]. Genetic analyses have identified various causes of ASD, such as fragile X syndrome, tuberous sclerosis, Rett syndrome, or chromosomal deletions and duplications detected by chromosomal microarrays [8]. In this patient, despite the absence of structural abnormalities in the oligonucleotide SNP chromosomal microarray, whole exome sequencing with CNV analysis revealed a mutation in the BICRA gene. This gene is associated with neurodevelopmental disorders but is rarely observed in ASD patients. Harraway et al. emphasize the clinical significance of advanced genetic testing, like whole exome sequencing, in uncovering potential causative mutations in ASD cases [9].

Notably, three SWI/SNF complex subdivisions, BAF, PBAF, and ncBAF, have been identified and associated with disorders, such as SSRIDD. Disorders like Coffin-Siris syndrome, characterized by intellectual disability, neurodevelopmental delay (including speech and motor delays), and coarse facial features, have been linked to BAF and PBAF complex mutations. Furthermore, the BICRA gene, exclusively involved in the ncBAF complex, adds to the complexity of understanding chromatin remodeling in neurodevelopmental disorders. This highlights the interaction between specific SWI/SNF complex subdivisions and their associated clinical manifestations, as revealed by Santen et al. in their study identifying mutations in AT-rich interaction domain 1B (ARID1B) associated with Coffin-Siris syndrome [10].

Barish et al. suggest that BICRA variants, presenting similarly to those seen in SSRIDD patients, such as moderate developmental delay, autism, intellectual disability, behavioral issues, and dysmorphic facial features, may represent a subgroup of the SWI/SNF complex, differing from Coffin-Siris syndrome by the lack of the fifth-digit/nail hypoplasia [11]. The patient described in this report was found to have a heterozygous mutation of the BICRA gene c.1246G>C (p.Ala416Pro) but lacks other symptoms typically associated with SWI/SNF complex mutations, such as vision abnormalities, dysmorphic facial features, and fifth-digit or nail hypoplasia.

Additionally, the BICRA gene has been associated with glioma tumor suppression. The study by Barish et al. discusses the functional significance of the BICRA gene and the ncBAF chromatin remodeling complex using zebrafish and fruit fly animal models. These findings provide insight into BICRA's role in neural development and related developmental disorders. By establishing the importance of BICRA in both human and fruit fly nervous system development, the study strengthens the connection between BICRA mutations and neurodevelopmental disorders [11]. These findings align with the broader understanding of BICRA's involvement in the etiology and manifestation of neurodevelopmental disorders, further validating the significance and importance of exploring said gene mutation.

## Conclusions

This case highlights the complexity of neurodevelopmental disorders and the importance of further understanding their underlying mechanisms. In order to fully understand BICRA's role in ASD, comprehensive research involving a broader patient population with similar phenotypic presentation should be conducted. Larger studies can provide a better understanding about ASD's nature by identifying target mutations within BICRA and exploring the extent of their genetic influence. Further efforts may involve cohort studies with affected groups to better understand the genetic basis of neurodevelopmental disorders. Additionally, exploring the potential for therapeutic development targeting BICRA mutations may lead to improved treatment options and better quality of life for patients with these mutations.
